# Oral anticoagulation on patients with atrial fibrillation: are we doing a good job?

**DOI:** 10.1080/07853890.2021.1896844

**Published:** 2021-09-28

**Authors:** Joana C. F. Lima, João Aguiar, Margarida Paixão Ferreira, Rita Calixto, Vera Cesário, José Vaz

**Affiliations:** a Serviço de Medicina Interna, Hospital José Joaquim Fernandes, Beja, Portugal; b Instituto de Investigação do Medicamento (iMED.ULisboa), Faculdade de Farmácia, Universidade de Lisboa, Lisboa, Portugal

## Abstract

**Introduction:**

Atrial fibrillation (AF) is one of the major causes of stroke and cardiovascular morbidity in the world [1]. Oral anticoagulation (OAC) with vitamin K antagonists (VKAs) or non-VKA oral anticoagulants (NOACs) reduces the risk of such events in AF patients [1–3]. Our aim was to evaluate if AF patients were correctly hypocoagulated and the prevalence of acute ischaemic stroke (IS) and acute transient ischaemic attack (TIA) among these patients.

**Materials and methods:**

A cross-sectional study was undertaken in a Portuguese hospital in Beja in the last three months. Patients (aged 18 years or older) with previous history of AF admitted to internal medicine ward were included. Data was extracted from medical charts, which included sociodemographic and clinical variables. To assess if patients were correctly medicated or in need for OAC, we calculated CHA_2_DS_2_-VASc (if <2: no need for OAC; if ≥2 (male) or ≥ 3 (female): need for OAC), assessed renal function (creatinine value; creatinine clearance using Cockcroft-Gault Equation), INR, and the type of OAC and doses. The informed consent of the subjects and acceptance of the study protocol by a local ethics committee has been obtained. Data analysis was performed using univariate statistics (IBM SPSS v.20.0).

**Results:**

A total of 150 patients were included, with a mean age of 81.8 ± 7.7 years old and 52.7% (*n* = 79) were female. Almost half of the sample was not on OAC (48.0%, *n* = 72). From the ones on OAC, 60.3% (*n* = 47) were on NOAC, with apixaban as the most prescribed drug (55.3%; *n* = 26), followed by rivaroxaban (31.9%; *n* = 15). A considerable proportion of patients was using warfarin (38.5%; *n* = 30). Almost 60% of the cases were incorrectly hypocoagulated, either due to lack of OAC prescription in patients that should be on that medication (80.7%; *n* = 71) or to incorrect dose (19.3%; *n* = 17). From patients who should be on OAC, 74.6% (*n* = 53) were aged 80 or older and 15.5% (*n* = 11) were admitted to internal medicine ward with acute IS (63.6%; *n* = 7) or acute TIA (36.4%; *n* = 4). Acute IS has been also registered in 4 patients (5.1%) correctly medicated (NOAC: *n* = 3; VKA: *n* = 1).

**Discussion and conclusions:**

Data suggest that almost half of the patients were not on OAC and 15.5% of these experienced an acute cerebrovascular event. Almost 75% of patients who should be on OAC were aged 80 or older. The considerable stroke risk without OAC often exceeds the bleeding risk even in the elderly, in patients with cognitive dysfunction, or in patients with frequent falls or frailty and these should not be the reasons for withholding, ending or do not initiate OAC.
